# Mouth-Level Intake of Benzo[a]pyrene from Reduced Nicotine Cigarettes

**DOI:** 10.3390/ijerph111111898

**Published:** 2014-11-18

**Authors:** Yan S. Ding, Jennye Ward, David Hammond, Clifford H. Watson

**Affiliations:** 1 Tobacco Analysis Laboratory, Division of Laboratory Sciences, National Center for Environmental Health, Centers for Disease Control and Prevention, 4770 Buford Highway, NE, Mailstop F-19, Atlanta, GA 30341, USA; E-Mails: vmc3@cdc.gov (J.W.); cow1@cdc.gov (C.H.W.); 2 School of Public Health and Health Systems, University of Waterloo, Waterloo, ON N2L 3G1, Canada; E-Mail: dhammond@uwaterloo.ca

**Keywords:** cigarette smoke, benzo[a]pyrene, mouth-level intake, cigarette filter, filter analysis, reduced nicotine cigarette, cotinine, smoking topography

## Abstract

Cigarette smoke is a known source of exposure to carcinogenic polycyclic aromatic hydrocarbons (PAHs), especially benzo[a]pyrene (BaP). Exposure to BaP in cigarette smoke is influenced by how a person smokes and factors, such as tobacco blend. To determine whether sustained use of reduced-nicotine cigarettes is associated with changes in exposure to nicotine and BaP, levels of BaP in spent cigarette filter butts were correlated with levels of BaP in cigarette smoke to estimate mouth-level intake (MLI) of BaP for 72 daily smokers given three progressively reduced nicotine content cigarettes. Urinary cotinine, a marker of nicotine exposure, and urinary 1-hydroxypyrene (1-HOP), a marker of PAH exposure, were measured throughout the study. Median daily BaP MLI and urine cotinine decreased in a similar manner as smokers switched to progressively lower nicotine cigarettes, despite relatively constant daily cigarette consumption. 1-HOP levels were less responsive to the use of reduced nicotine content cigarettes. We demonstrate that spent cigarette filter butt analysis is a promising tool to estimate MLI of harmful chemicals on a per cigarette or per-day basis, which partially addresses the concerns of the temporal influence of smoking behavior or differences in cigarette design on exposure.

## 1. Introduction

Tobacco smoke is a leading cause of lung cancer and related preventable diseases [1]. Among the thousands of chemical constituents in cigarette smoke, benzo[a]pyrene (BaP), an International Agency for Research on Cancer (IARC) human group 1 carcinogen, is one of the most potent carcinogens [2,3]. Delivery of BaP in mainstream smoke is affected by the blend of tobacco in the cigarette filler (flue-cured or burley), cigarette filter type (e.g., percent ventilation) and how the cigarette is smoked (puff volume and puff frequency) [4]. Estimating and monitoring human exposure to BaP from cigarette smoke is an important public health challenge. Because polycyclic aromatic hydrocarbons (PAHs), such as BaP, are formed from the incomplete combustion of organic matter, there are multiple exposure sources (smoking, cooking, automobile exhaust,* etc.*). There are also technical difficulties in measuring the metabolites of BaP in human specimens. Instead, surrogate biomarkers, such as urinary1-hydroxypyrene (1-HOP), a metabolite of pyrene, are commonly measured to estimate BaP exposure [5].

Other than measuring surrogate compounds, indirect approaches have been developed to estimate BaP exposure [6]. We previously developed and published a method involving chemical analysis of spent cigarette filter butts [7]. During smoking, cigarette filters trap a significant portion of mainstream smoke constituents, including BaP. The amount trapped in the cigarette filter is proportional to the total volume of mainstream smoke drawn into the mouth. By measuring the amount of BaP in a spent filter as a function of mainstream smoke BaP, the smoker’s mouth-level intake from a cigarette can be determined [7]. Standardized machine smoking measurements quantify how much of a toxicant are present in smoke, but are known to be poor predictions of human intake and the resulting exposure [6]. Biomarkers of exposure are effective measures of an individual’s exposure, but generate a “time-averaged” exposure based on all recent exposures. The spent filter analysis was developed as an alternative, non-invasive means to investigate smoking intake and patterns that accounts for variations in smoking behavior, such as larger puffs, smaller puffs and rapid smoking. Spent filter analysis can be used to determine the mouth-level intake of a toxicant in cigarette smoke on a per puff, per cigarette or cigarettes per day basis [6,7,8].

Over the last decade, research using cigarette filter-based assays as proxies for toxicant exposure and smoking behavior has been continuously growing [6]. Existing data indicate that filter nicotine assays show excellent correlations to nicotine biomarker measurements [8]. The more recent research focus is on finding linkages between filter-based assays and biomarkers of exposure to constituents other than nicotine [6]. Estimating mouth-level intake and comparing with surrogate biomarkers can provide valuable evidence for such linkages and help establish source attribution for environmental pollutants on an individual or population basis. In addition, it is important to characterize exposure to toxicants in smokers when provided with cigarettes that differ in design features or in levels of important constituents, such as nicotine. Characterizing exposure among smokers is complicated by conscious or unintentional changes in smoking behavior when smoking a cigarette that differs in nicotine content from their usual brand. 

The filter analysis method for estimating mouth-level BaP intake is applied in this current work by analyzing discarded cigarette filters collected from smokers participating in a clinical study designed to investigate human smoking behavior with different product types. This study investigated how adult smokers respond when switched to progressively lower nicotine content cigarettes (Quest^®^ cigarettes) in an un-blinded trial. Nicotine is the primary addictive component in tobacco. At present, all commercial cigarette brands contain ample nicotine to promote and sustain addiction. Previous research demonstrates that smokers can increase the intensity of their smoking behavior to compensate for modest differences in the level of nicotine present in cigarette smoke [9,10,11]. However, it remains unclear whether smokers will engage in similar “compensatory” behavior in response to substantial reductions in nicotine content that go well beyond the small variations in levels of nicotine in the smoke of conventional cigarette brands. Despite a few studies [12,13,14], there is limited evidence as to whether smokers compensate for the reduced nicotine by smoking these cigarettes more intensely and whether changes in smoking behavior will affect smokers’ exposure to other important toxicants in cigarette smoke. 

The objectives of this study were to determine whether: (1) changes occurred in smokers’ smoking intensity when switched to reduced-nicotine cigarettes; (2) trends existed of the overall daily mouth-level BaP intake from naturalistic cigarette smoking throughout the collection periods without controlling diet or environmental exposures; and (3) correlations exist with traditional urine biomarkers of cigarette smoke exposure. We looked into the ability of spent filter analysis to characterize the mouth-level intake of a prevalent environmental pollutant that provides evidence of changes in smoker behavior that can accompany changing to a cigarette brand that differs in design or composition. We also discuss the reliability of using mouth-level BaP intake to assess a smoker’s exposure compared to using urinary 1-HOP, a common surrogate exposure biomarker [15].

## 2. Experimental Section

### 2.1. Summary of Study Protocol

In the current study, 72 adult Canadian smokers completed an un-blinded trial of reduced nicotine content cigarettes. Smokers’ profile included being between 18–65 years old, using a minimum of five Canadian brand cigarettes per day, having no intention to quit and not making use of any nicotine replacement therapy or other tobacco products. Participants completed a 7-day baseline period during which they smoked their usual cigarette brand. Participants then smoked Quest^®^ cigarette brands (Quest^®^ 1, Quest^®^ 2 and Quest^®^ 3) with progressively lower nicotine levels (6 mg, 3 mg, and 0.05 mg nicotine content). Each Quest^®^ brand was smoked for a 7-day period, for a total of 3 consecutive weeks. Participants were asked to collect cigarette spent filters on Day 2 and Day 7 of each period. They reported for a clinic visit each week to submit cigarette spent filters, at which time, validated measures of nicotine dependence and withdrawal, patterns of smoking behavior and a urine specimen of the visit day were collected for that 7-day period. The whole study trial lasted from May 2009, until June 2010. The study protocol was reviewed and approved by the University of Waterloo office of research ethics. All subjects gave informed written consent before participating in the study. CDC involvement only consisted of receiving and testing spent cigarette filters and did not constitute engagement in human subject research. 

### 2.2. Standards, Reagents and Materials

BaP was purchased from Cambridge Isotope Laboratories (Andover, MA, USA). Nicotine, acetonitrile, acetone and cyclohexane were purchased from Sigma (St. Louis, MO, USA) and were HPLC-grade. Isotopically-labeled nicotine was purchased from Toronto Research Chemicals Inc. (North York, ON, Canada). Cambridge filter pads (CFP, 44-mm glass fiber) were obtained from Whatman (Maidstone, UK). Blank cellulose acetate cigarette filters were obtained from Filtrona (Richmond, VA, USA). Quest^®^ cigarettes were purchased from a retail outlet in New York. Other commercial cigarettes were purchased from retail stores in Canada.

### 2.3. Measurement of Cigarette Physical Properties and Machine-Smoked Emission

#### 2.3.1. Cigarette Physical Properties

A Cerulean C2 instrument (Milton Keynes, UK) was used to measure filter ventilation. Cigarette length, weight and filter length were measured manually and recorded for all of the cigarette brands turned in by participants. 

#### 2.3.2. Machine-Smoke Regimens and Smoke Sample Collection

Cigarettes and CFPs were conditioned at 22 °C and 60% relative humidity for at least 24 h prior to smoking, according to ISO 3308 [16]. Cigarettes were smoked either to a specific length or to the marked length of the filter overwrap (tipping) plus 3 mm using a Cerulean ASM500 16-port or a Cerulean SM450 20-port smoking machine (Table 1). Individual cigarettes were smoked per CFP for each individual sample for the correlation between filter butts and the mainstream smoke total particulate matter (TPM). TPM generated under different smoking regimens was collected on individual CFPs, and the corresponding cigarette filters were collected. After smoking, CFPs and cigarette filters were quantitatively analyzed for BaP content.

**Table 1:** Machine smoking regimens used to establish correlation relations between benzo[a]pyrene (BaP) in the spent cigarette filter butts and mainstream smoke deliveries.

Regimen	Puff Volume (mL)	Interval (Second)	Puffs or Smoked Length	Filter Vent Holes
ISO	35	60	2 at the lighting end	Open
ISO	35	60	Butt mark as defined in text	Open
Canadian Intense	55	30	Butt mark as defined in text	Closed
Intense 1	65	20	Butt mark as defined in text	Closed
Intense 2	75	30	Butt mark as defined in text	Closed
Intense 3	70	10	Butt mark as defined in text	Closed

In addition to BaP, mainstream smoke emission nicotine levels from the Quest^®^ cigarettes and commercial cigarettes were measured on a per brand basis. Cigarettes were smoked by a smoking machine using ISO and Canadian intense regimens. Each smoking condition was repeated three times. After smoking, CFPs were quantitatively analyzed for nicotine content.

#### 2.3.3. Analysis of BaP from Machine-Smoked CFPs and Cigarette Filters

A previously published method was used in the preparation of smoke samples from CFPs and machine-smoked cigarette filters [7]. Briefly, BaP on CFPs was extracted by cyclohexane and cleaned up by solid phase extraction (SPE). To extract BaP from each filter, a portion (10 mm) was removed from the mouth end, stripped of wrapping paper, dissolved in acetone and subjected to the same SPE clean up as the CFPs. Belmont Silver, one of the subjects’ usual brands, and Quest^®^ 2 brands have two-part filters with mouth-end portions shorter than 10 mm. Therefore, BaP filter analyses for Belmont Silver and Quest^®^ 2 involved 8- and 9-mm filter portions, respectively. Samples were then analyzed by an Agilent 1200 high-performance liquid chromatography coupled with a fluorescence detector (HPLC-FLD) (Agilent Technologies, Wilmington, DE, USA). 

#### 2.3.4. Analysis of Nicotine from Machine-Smoked CFPs

Machine-smoked mainstream smoke nicotine delivery was analyzed based on the previously published method [17]. After smoking, each CFP was spiked with isotopically-labeled nicotine internal standard solutions; this was followed by solvent extraction. An aliquot was analyzed by HPLC coupled with an API 5500 triple quadruple mass spectrometer (HPLC-MS/MS) (AB Sciex, Foster City, CA, USA) to obtain nicotine levels in each brand.

### 2.4. Measurement of BaP from Spent Cigarette Filter Samples and Urinary Cotinine and 1-HOP

#### 2.4.1. Analysis of BaP from Spent Cigarette Filter Butts

The entire spent cigarette filter butt inventory for this study includes more than 10,000 filters from 72 participants. The filter butts were shipped to the CDC in 2-mL Cryovials and stored at −70 °C until analyzed. A subset of filters from every participant (16 per participant, ~1200 total) was selected. The subset represented each of the four collection periods where participants subsequently smoked their usual commercial brand, then sequentially smoked the progressively lower nicotine series cigarettes: Quest^®^ 1, Quest^®^ 2 and Quest^®^ 3. The BaP from participants’ spent filters were extracted and analyzed by the same preparation procedure used for the machine smoked cigarette filters.

#### 2.4.2. Analysis of Urinary Cotinine and 1-HOP

Urinary cotinine (the main metabolite of nicotine) and 1-HOP were measured and reported by Labstat International ULC, Canada.

### 2.5. Mathematic Calculation and Statistical Analysis

#### 2.5.1. Brand Correlation Data

Cigarettes from each brand (36 commercial brands and 3 Quest^®^ brands) were machine smoked using variations of standard smoking machine regimens (Table 1). The resulting mainstream smoke deliveries of BaP were used to establish the correlation regression models using linear least squares regression (Excel) to relate levels of BaP in the spent cigarette filters and levels from CFPs. The mouth-level intake of BaP per cigarette (BaP/cigarette) was estimated using the measured spent cigarette filter BaP levels in the brand-specific regression model equation. Total mouth-level BaP intake per day was calculated by multiplying the average BaP/cigarette intake by the number of cigarette consumed per day for each participant.

#### 2.5.2. Statistical Analysis

Total mouth-level BaP intakes of all participants from four collection periods were compared by calculating the 5th, 25th, 50th, 75th and 95th percentiles of each period. The percentiles are visually displayed as a box plot (Sigma Plot). The differences in the median values between each two periods were tested by the Mann–Whitney rank sum test (Sigma Plot) for statistical significance. The same analyses (box plot and rank sum test) were also performed on urinary cotinine and 1-HOP data collected from the four collection periods.

## 3. Results

### 3.1. Cigarette Physical Properties, Machine-Smoked Emission Data and Brand Correlation Parameters

#### 3.1.1. Physical Properties

The 72 participants smoked a total of 36 different commercial cigarette brands and three Quest^®^ cigarette brands (Table 2). Among them, three participants did not have valid data from the first collection period, so we did not use these data. Therefore, data analyzed for the first period (subjects’ usual brand) only contained 69 subjects. Tip ventilation of these cigarettes ranged from 0 to 50%. Cigarette weights were between 0.70 and 0.90 grams. Most cigarette lengths (69%) were king size (83 ± 1mm); two brands were approximately 100 mm in length; and the remaining brands were either 71 or 72 mm in length. All of the cigarettes were the same diameter. 

Most cigarette brands had cellulose acetate filters, except for two Belmont brands. They contained a two-part filter assembly, made of cellulose acetate (10 mm for regular and 8 mm for silver) and a “Dalmatian” style charcoal (12 mm for regular and 11 mm for silver) in order from the mouth-end moving up the rod. All three Quest^®^ cigarettes had filter ventilation (45%, 22% and 39% for Quests^®^ 1, 2 and 3, respectively). Quest^®^ 2 and 3 cigarettes also contained two-part filters. Quest^®^ 2 had a cellulose acetate (9 mm) and grey baffled filter (16 mm), whereas Quest^®^ 3 had a cellulose acetate (16 mm) and white baffled filter (9 mm) (both in order from mouth-end moving up the rod) (Table 2). 

**Table 2:** Cigarette physical properties of all participants’ usual brands and the Quest^®^ brands.

Cigarette Brand	Filter Ventilation Level (%)	Cigarette Weight (g)	Cigarette Length (mm)	Filter Length (mm)
Belmont Regular	0	0.90	83	22 (10 + 12) *
Belmont Silver	21	0.82	71	19 (8 + 11) *
Benson & Hedges Silver Menthol	28	0.96	99	27
Canadian Classics Reg. King	7	0.94	83	22
Canadian Classics Regular	0	0.78	71	19
Canadian Classics Silver King	14	0.92	83	22
Canadian Classics White King	27	0.92	83	22
Du Maurier Distinct Regular	19	0.93	100	25
Du Maurier Distinct King	25	0.88	83	21
Du Maurier Premier King	36	0.91	84	20
Du Maurier Premier Regular	34	0.78	72	17
Du Maurier Prestige	26	0.88	82	20
Du Maurier Regular King	0	0.87	83	20
Du Maurier Regular	12	0.76	71	17
Du Maurier Special	21	0.90	83	20
Export A Green	0	0.81	71	17
Export A Extra Smooth	22	0.77	71	17
Export A Ultra Smooth	27	0.87	83	22
John Player Standard Blue	0	0.77	72	16
John Player Standard Blue King	0	0.87	83	20
John Player Standard Sliver King	20	0.84	83	20
Macdonald Special	0	0.85	84	21
Next Blue	7	0.89	82	22
Number 7 Blue	22	0.90	82	22
Number 7 Regular	10	0.89	83	22
Peter Jackson Full King	0	0.87	82	20
Peter Jackson Full Regular	0	0.79	72	17
Peter Jackson Select King	16	0.89	82	20
Peter Jackson Select Regular	16	0.81	72	17
Peter Jackson Smooth King	31	0.92	83	19
Player’s Original King	11	0.86	82	20
Player’s Rich Regular	8	0.74	72	17
Player’s Rich King	12	0.85	84	20
Player’s Smooth King	26	0.87	82	20
Viceroy Blue	19	0.89	82	20
Vogue Slims	50	0.73	82	21
Quest^®^ 1	45	0.82	83	25
Quest^®^ 2	22	0.89	83	25 (9 + 16) *
Quest^®^ 3	39	0.87	83	25 (16 + 9) *

* The number in the parentheses indicates two filter segments. Quest^® ^is the registered trademark of Vector Tobacco Ltd. (Vector Tobacco Inc., Mebane, NC, USA) Canadian brands are registered trademarks of the respective Canadian tobacco companies.

#### 3.1.2. Machine-Smoked Emission Data

Mainstream smoke nicotine and BaP under two smoking regimens (ISO and Canadian intense) were measured and reported (Table 3). Most brands were measured in triplicate, except for four brands with limited cigarette quantities. Belmont Regular, Du Maurier Prestige and Quest^®^ 1 had duplicate analyses; Du Maurier Regular King had a single analysis. Mainstream smoke nicotine yields were approximately 1 mg per cigarette under the ISO regimen for most brands. An exception was Vogue Slims, which had approximately 0.4 mg nicotine per cigarette (ISO). 

Mainstream smoke nicotine (ISO) from Quest^®^ 1 and 2 was 0.7 and 0.3 mg per cigarette, respectively. Mainstream smoke nicotine from Quest^®^ 3 cigarettes was below the reportable range (reported as <0.0025 mg). Mainstream smoke BaP was higher in most Canadian cigarettes (constructed with bright tobacco) than in Quest^®^ 1, 2 and 3 (constructed with a blend of tobacco types) (Table 3). 

**Table 3:** Mainstream smoke deliveries of nicotine and BaP from the cigarettes in this study. ISO and Canadian intense regimens were tested.

Cigarette Brand	ISO		Canadian Intense	
	Nicotine (mg/cigarette)	BaP (ng/cigarette)	Nicotine (mg/cigarette)	BaP (ng/cigarette)
Belmont Regular	0.8	9.0	1.9	18.1
Belmont Silver	1.0	7.2	2.2	13.7
Benson & Hedges Silver Menthol	1.2	8.3	3.1	16.2
Canadian Classics Reg. King	1.3	12.1	2.7	19.4
Canadian Classics Regular	1.3	9.4	2.5	20.4
Canadian Classics Silver King	1.2	7.6	2.4	16.7
Canadian Classics White King	0.9	6.7	2.4	16.6
Du Maurier Distinct Regular	1.2	12.9	2.4	22.5
Du Maurier Distinct King	1.3	8.8	3.0	19.1
Du Maurier Premier King	0.9	7.3	2.4	16.4
Du Maurier Premier Regular	0.8	6.0	2.1	13.8
Du Maurier Prestige	1.1	8.6	2.2	24.2
Du Maurier Regular King	1.1	13.4	2.6	22.7
Du Maurier Regular	1.2	9.1	2.3	14.9
Du Maurier Special	1.3	8.8	2.6	18.2
Export A Green	1.4	12.3	2.3	20.6
Export A Extra Smooth	1.0	8.4	2.2	16.7
Export A Ultra Smooth	1.0	7.6	2.3	19.6
John Player Standard Blue	1.3	7.9	2.2	20.5
John Player Standard Blue King	1.1	11.9	2.3	21.4
John Player Standard Sliver King	1.2	10.9	2.3	23.4
Macdonald Special	0.8	12.0	2.2	22.7
Next Blue	1.2	10.1	2.7	19.1
Number 7 Blue	1.1	8.8	2.9	20.7
Number 7 Regular	1.3	9.9	2.7	18.7
Peter Jackson Full King	1.1	12.0	2.5	21.5
Peter Jackson Full Regular	1.2	10.5	2.3	19.8
Peter Jackson Select King	1.1	13.0	2.6	22.1
Peter Jackson Select Regular	1.2	10.4	2.6	17.8
Peter Jackson Smooth King	0.9	7.3	2.3	15.3
Player’s Original King	1.4	16.0	2.9	23.2
Player’s Rich Regular	1.0	10.9	2.3	16.1
Player’s Rich King	1.1	9.4	2.4	20.4
Player’s Smooth King	0.9	7.5	2.5	15.7
Viceroy Blue	1.1	9.6	2.6	16.9
Vogue Slims	0.4	3.2	1.8	10.2
Quest^®^ 1	0.7	6.1	1.9	22.8
Quest^®^ 2	0.3	3.1	0.8	10.8
Quest^®^ 3	<0.0025	1.8	0.1	9.2

Cigarettes were smoked according to ISO 3308. Mainstream total particulate matter was collected on a Cambridge filter pad (CFP).

#### 3.1.3. Brand Correlation Parameters

Difference in the design features of the cigarette brands required establishing the BaP correlation for each brand individually between CFPs and filters (Table 4). Excellent linearity was observed for BaP between mainstream smoke yield and filter tips from the three reduced-nicotine cigarettes and all of the Canadian brands. The regression parameters (slope, intercept and correlation coefficient (*r*^2^)) were as follows: slopes ranged from 2.1 (Quest^®^ 3) to 7.6 (Quest^®^ 2); the *r*^2^ values for the majority of the Canadian brands were greater than or equal to 0.90, except for four brands, where *r*^2^ ranged from 0.82 to 0.88. The number of participants who smoked each Canadian brand ranged from one to six. None of the 72 participants declined the three Quest^®^ brands.

### 3.2. Mouth-Level Intake per Cigarette and Smoking Intensity

We measured BaP from the 10-mm mouth-end filters and estimated the mouth-level intake of BaP using the brand-specific correlation regression models. To establish a means to quantify and characterize smoking “intensity” via the amount of BaP trapped in the spent filter butts, we set a cut-off level for spent filter BaP that indicated that the participant’s smoking behavior exceeded the top levels of BaP generated when the cigarettes were machine-smoked with the Canadian intense regimen. We were then able to characterize smoking intensity levels from every participant during each seven-day period to partially address the question of the extent of compensatory smoking behavior. We observed differences in smoking intensity throughout the four smoking periods. During the initial baseline period when participants smoked their usual brands, results from 57% of spent filters indicated that subjects smoked cigarettes more intensely than the Canadian intense regimen. When participants switched to Quest^®^ 1 cigarettes, only 21% of spent filters were smoked more intensely than the Canadian intense regimen. The majority of participants, 79% (Quest^®^ 1) and 70% (Quest^®^ 2), smoked the reduced-nicotine cigarettes with less intensity than the Canadian intense regimen. Further, when switched to the ultra-low nicotine delivery Quest^®^ 3, only 4% of cigarettes were smoked more intensely than the cigarettes that were machine-smoked with Canadian intense regimens (Table 5). 

**Table 4:** Relations between BaP measured in mainstream smoke and spent filter butts. A correlation regression model for each brand was established using linear regression.

Cigarette Brand	Slope	Intercept	*r*^2^
Belmont Regular	5.2	−0.4	0.94
Belmont Silver	3.8	−0.1	0.99
Benson & Hedges Silver Menthol	3.3	0.6	0.82
Canadian Classics Reg. King	6.5	−2.5	0.96
Canadian Classics Regular	4.4	−0.4	0.96
Canadian Classics Silver King	5.0	−2.4	0.91
Canadian Classics White King	4.7	−2.5	0.97
Du Maurier Distinct Regular	4.5	0.9	0.96
Du Maurier Distinct King	2.7	1.1	0.96
Du Maurier Premier King	2.3	1.3	0.94
Du Maurier Premier Regular	2.9	−0.9	1.00
Du Maurier Prestige	3.4	−2.6	0.99
Du Maurier Regular King	3.0	3.0	0.96
Du Maurier Regular	2.6	0.0	0.99
Du Maurier Special	3.5	−1.2	1.00
Export A Green	6.5	−3.4	0.97
Export A Extra Smooth	3.0	−0.1	0.98
Export A Ultra Smooth	3.1	0.2	0.99
John Player Standard Blue	5.2	−7.9	0.87
John Player Standard Blue King	3.3	−0.6	0.96
John Player Standard Sliver King	3.2	3.0	0.88
Macdonald Special	5.4	1.1	0.95
Next Blue	6.3	−5.1	0.86
Number 7 Blue	6.3	−3.5	0.96
Number 7 Regular	5.8	−1.2	0.92
Peter Jackson Full King	4.2	−3.1	0.98
Peter Jackson Full Regular	3.5	−1.0	1.00
Peter Jackson Select King	3.9	−2.7	0.98
Peter Jackson Select Regular	3.3	−0.6	1.00
Peter Jackson Smooth King	2.6	0.0	1.00
Players Original King	5.3	−0.3	0.97
Players Rich Regular	3.2	0.9	0.93
Player’s Rich King	3.3	−0.8	0.97
Player’s Smooth King	3.8	−1.4	0.98
Viceroy Blue	3.0	1.7	0.96
Vogue Slims	3.0	0.1	0.99
Quest^®^ 1	4.9	−1.7	0.90
Quest^®^ 2	7.6	0.3	0.95
Quest_®_ 3	2.1	1.7	0.90

**Table 5:** Percentage of subjects’ spent filter butts that exhibited a higher yield of BaP than the butts from machine-smoked ones with the Canadian intense regimen.

Cigarette Brand	Above Canadian Intense
Smoker’s usual brand	57%
Quest^®^ 1	21%
Quest^®^ 2	30%
Quest^®^ 3	4%

To observe whether participants smoked more cigarettes as they switched from their usual brands to Quest^®^ brands, we counted each subject’s daily cigarette consumption. The average cigarette per day (CPD) was 14.8 ± 6.4 when participants smoked their usual brands. CPDs for Quest^®^ 1, 2 and 3 periods were 14.8 ± 6.7, 15.8 ± 7.6 and 15.0 ± 9.4, respectively. 

### 3.3. Total Mouth-Level BaP Intake per Day and Urinary Biomarkers

**Figure 1:**
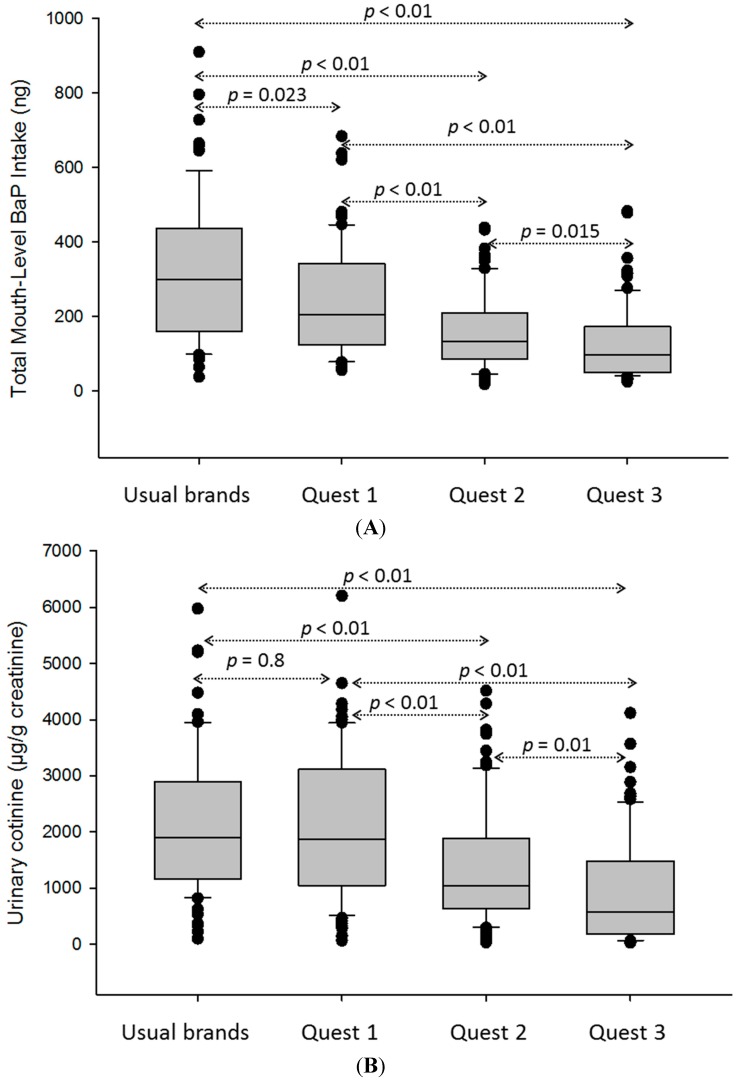

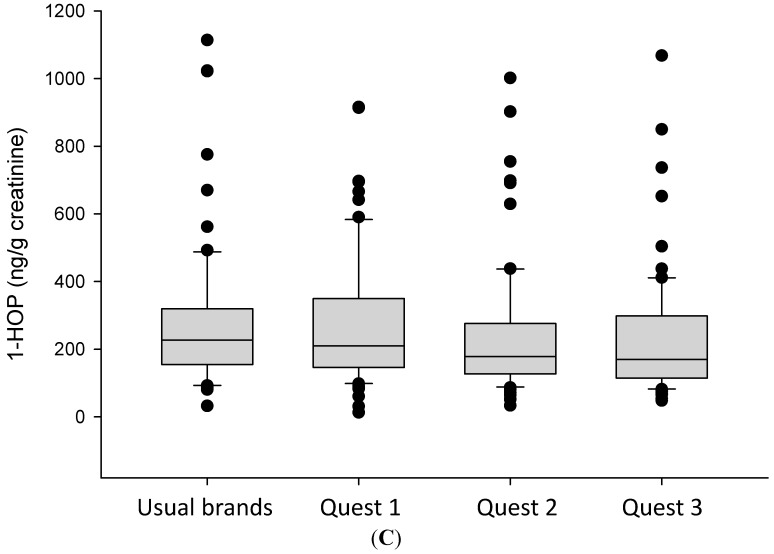
Comparison of daily mouth-level intake of BaP and other biomarkers over the four smoking periods. (**A**) Total mouth-level intake of BaP; (**B**) urinary cotinine; (**C**) urinary 1-hydroxypyrene (1-HOP). Statistically significant values were displayed between each two collection periods for BaP and cotinine. There is no statistical significance between each of the two collection periods for 1-HOP.

The median total mouth-level BaP intake decreased as participants switched to the reduced-nicotine Quest^®^ cigarettes (Figure 1A, median ranging from 298 to 96 ng). There were statistically significant differences between each collection period. We applied the same analyses to the biomarkers (urinary cotinine and 1-HOP) from the same group (Figure 1B,C, respectively). Box plot results indicated a similar decreasing trend of median urinary cotinine (Figure 1B, median ranging from 1896 to 579 µg/g creatinine); there were statistically significant differences between each collection period, except between usual brands and Quest^®^ 1. Median 1-HOP did not change over the four periods when smokers switched to Quest^®^ cigarettes (Figure 1C, median ranging from 227 to 170 µg/g creatinine); also, there were no statistically significant differences in 1-HOP between each collection period.

## 4. Discussion

There were 72 adult Canadian smokers participating in this study, and the subjects smoked a wide variety of Canadian brand cigarettes. Most brands have several sub-brands encompassing a wide range of filter tip ventilations, rod lengths and filter lengths and types (Table 2). Canadian cigarettes contain almost exclusively bright tobacco [18]; whereas Quest^®^ cigarettes contain “U.S. blended” tobacco, which typically contains approximately 35% bright, 30% burley, 20%–30% reconstituted tobacco leaf, as well as a smaller amount of air-cured and oriental tobaccos [18]. Most of the participant’s usual brand of cigarettes had a standard cellulose acetate filter, except the two Belmont brands and Quest^®^ 2 and Quest^®^ 3, which contained two filter segments. Despite the differences in physical properties, the machine-generated mainstream smoke nicotine deliveries were similar (around 1 mg per cigarette for the ISO regimen) among all of the participants’ usual brands. Vogue Slims have low emission data (Table 3) due to their high filter ventilation (Table 2). However, when the Vogue Slims brand was smoked under the Canadian intense regimen, mainstream smoke emission data were comparable to other brands smoked under the intense regimen. Quest^®^ cigarettes delivered lower mainstream smoke nicotine than the participants’ usual brands under both ISO and Canadian intense smoking conditions (Table 3). Quest^®^ cigarettes are manufactured with low nicotine tobacco and increased filter ventilation and filter efficiency. 

The spent filter analysis method has two main advantages compared to traditional exposure metabolites or surrogate metabolite analyses. One of those advantages is the ability to estimate mouth-level BaP intake on a per cigarette or cigarette per day basis in a manner that accounts for the range of naturalistic smoking behaviors. More than 50% of the spent filters from the participants’ usual brands indicated that participants smoke more intensely than the smoking machine parameters of the Canadian intense regimen (Table 5). This observation supports the conclusion by Hammond* et al.* [10] that the maximum delivery generated under Canadian intense machine smoking conditions may be closer to an average human smoking delivery than the maximum in what smokers can obtain from a cigarette [10]. When smokers switched to the low nicotine content Quest^®^ cigarettes, the percentage of the spent filters indicating more intensive smoking than the Canadian intense regimen dropped to below 30% (Table 5). This indicates a low to moderate attempt to compensate for lower nicotine delivery in smoke by smoking more intensely when provided with the low nicotine Quest^®^ 1 and 2 cigarettes. With daily cigarette consumption remaining constant, participants may have smoked less intensely. Another explanation could be that Canadian smokers did not like the taste of the American blended Quest^®^ cigarettes. Interestingly, when switching from Quest^®^ 2 to Quest^®^ 3, more than 95% of the spent filters indicate lower smoking intensity than the Canadian intense regimen (Table 5). Because this study was an un-blinded trial, participants knew they were smoking either their usual brand or low nicotine cigarettes (including the nicotine level of Quest^®^ 3). It could be that the participants did not increase the intensity of their smoking behavior because they knew or suspected they would be unsuccessful in obtaining more nicotine. This observation agrees with a previous study of reduced-nicotine cigarettes, that smoking a 0.3-mg cigarette, but not a 0.05-mg cigarette, was associated with compensatory smoking behaviors [19]. Furthermore, awareness of the unique low nicotine nature of Quest^®^ cigarettes may have encouraged a social desirability bias in participants and discouraged compensation behavior that might occur outside the conditions of the experiment. Our observations of participants’ smoking intensity changes and constant daily cigarette consumption agree with a previous review stating that changing the puff volume or frequency is the most probable mechanism of compensational smoking and that changing the number of cigarettes does not appear to be a common mechanism of compensational smoking [20].

Another advantage of the spent filter approach is the ability to provide an alternative estimation of BaP intake from cigarette smoke rather than the commonly used 1-HOP surrogate biomarker. Over the four collection periods, the median daily mouth-level BaP intake from the spent filter analysis showed a decreasing trend as participants switched from their usual brands to Quest^®^ brands (Figure 1A). The decrease partially results from a lower BaP smoke yield in Quest^®^ 2 and 3 compared to Quest^®^ 1 (Table 3) and the less intensive smoking observed as the nicotine content is reduced (Table 5). This trend mirrors decreases in urinary cotinine (creatinine adjusted) levels from the same group. The cotinine results support the conclusion that compensatory smoking behavior to obtain more nicotine from Quest^®^ 3 cigarettes was not occurring. However, we observed that the trend of urinary 1-HOP (creatinine adjusted) remained steady over the four collection periods. The lack of agreement between 1-HOP and other exposure measures is consistent with previous studies indicating that 1-HOP remains stable when smokers switch to reduced nicotine content cigarettes [13,14]. Urine 1-HOP has been used in several reduced-nicotine cigarette studies as a surrogate measure of exposure to PAHs in tobacco smoke [13,14]. Urine 1-HOP measurements reflect PAH exposures other than smoking, such as through ingestion of cooked food and inhalation of automobile exhaust or other pollution sources. We noted a small amount of noncompliance among study participants indicated by a small number of participants’ usual brand butts found during the Quest^®^ cigarette smoking periods. If noncompliance was underestimated and a significant numbers of smokers smoked their usual brands during the Quest^®^ cigarette smoking periods or if they had substantial non-tobacco exposures to PAHs, the 1-HOP results could be biased. However, urine cotinine values reflecting nicotine intake over all phases of the study showed no evidence of substantial expected exposure to higher nicotine intake from cigarette smoke. The median 1-HOP level in our study is about twice that of the National Health and Nutrition Examination Survey reported values from U.S. smokers [5]. However, Canadian brand cigarettes contain bright tobacco, which generates higher smoke levels of PAHs than cigarettes constructed with U.S. blended tobacco. Therefore, it is likely that the tobacco type, and possibly other unforeseen factors, such as filter efficiency, or other non-smoking exposures to PAHs were occurring and affecting participants’ body burden of PAHs. 

## 5. Conclusions

Our results are comparable to previous reports suggesting that smokers will try to compensate when smoking cigarettes with modest nicotine reduction. However, for the lowest nicotine level Quest^®^ 3 brand, compensatory smoking behavior was not apparent. Cigarette filter analysis in general and, specifically, analysis of a prevalent environmental pollutant, such as BaP, are resources to estimate the mouth-level intake of toxicants of concern, both on a per-cigarette or per-day basis, in a manner that provides valuable information on how product characteristics can influence exposure assessments. In addition to spent filter analysis of nicotine, tobacco-specific nitrosamines and solanesol [6,20,21,22], this analysis fills a gap by specifying BaP exposure at the individual cigarette level, and it is an excellent tool for monitoring the impact of product alterations on human smoking behavior. The results also demonstrate the possibility of applying this technique to other markers of tobacco product exposure. 
